# Two M-T hook residues greatly improve the antiviral activity and resistance profile of the HIV-1 fusion inhibitor SC29EK

**DOI:** 10.1186/1742-4690-11-40

**Published:** 2014-05-27

**Authors:** Huihui Chong, Zonglin Qiu, Jianping Sun, Yuanyuan Qiao, Xingxing Li, Yuxian He

**Affiliations:** 1 MOH key Laboratory of Systems Biology of Pathogens and AIDS Research Center, Institute of Pathogen Biology, Chinese Academy of Medical Sciences & Peking Union Medical College, Beijing 100730, P. R. China

**Keywords:** HIV-1, Fusion inhibitor, M-T hook structure, Resistance

## Abstract

**Background:**

Peptides derived from the C-terminal heptad repeat (CHR) of HIV-1 gp41 such as T20 (Enfuvirtide) and C34 are potent viral fusion inhibitors. We have recently found that two N-terminal residues (Met115 and Thr116) of CHR peptides form a unique M-T hook structure that can greatly enhance the binding and anti-HIV activity of inhibitors. Here, we applied two M-T hook residues to optimize SC29EK, an electrostatically constrained peptide inhibitor with a potent anti-HIV activity.

**Results:**

The resulting peptide MT-SC29EK showed a dramatically increased binding affinity and could block the six-helical bundle (6-HB) formation more efficiently. As expected, MT-SC29EK potently inhibited HIV-1 entry and infection, especially against those T20- and SC29EK-resistant HIV-1 variants. More importantly, MT-SC29EK and its short form (MT-SC22EK) suffered from the difficulty to induce HIV-1 resistance during the *in vitro* selection, suggesting their high genetic barriers to the development of resistance.

**Conclusions:**

Our studies have verified the M-T hook structure as a vital strategy to design novel HIV-1 fusion inhibitors and offered an ideal candidate for clinical development.

## Background

**Figure 1:**
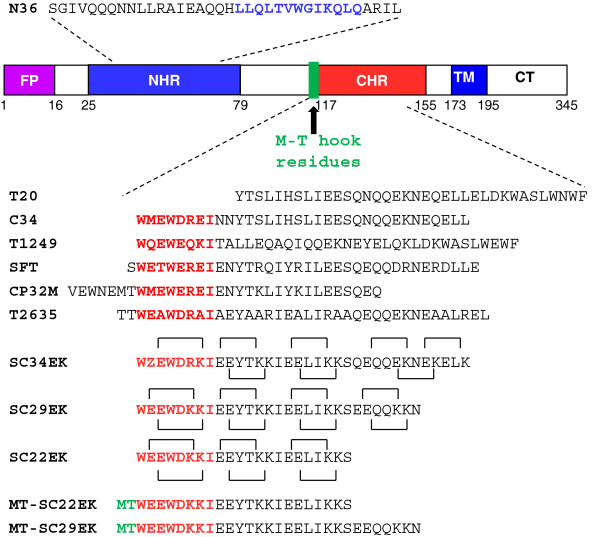
**Schematic diagram of HIV-1 gp41 and sequences of NHR or CHR-derived peptides.** The gp41 numbering of HIV-1_HXB2_ is used. FP, fusion peptide; NHR, N-terminal heptad repeat; CHR, C-terminal heptad repeat; TM, transmembrane domain; CT, cytoplasmic tail. The sequences corresponding to the NHR pocket region are marked in blue; The sequences corresponding to the CHR pocket-binding domain are marked in red; The position and sequence for the M-T hook structure are marked in green. Z in the SC34EK sequence indicates an artificial amino acid, norleucine. The possible electrostatic interactions are indicated by lines and correlating amino acids.

Entry of HIV-1 into target cells is mediated by its trimeric envelope (Env) glycoprotein gp120/gp41 complex and includes two major steps [1,2]. First, the surface subunit gp120 binds sequentially to the cell receptor and a coreceptor (CCR5 or CXCR4). Second, the transmembrane subunit gp41 (Figure 1) inserts its fusion peptide (FP) into the cell membrane and then packs its three C-terminal heptad repeat region (CHR) onto three N-terminal heptad repeat region (NHR) to form a six-helical bundle (6-HB) structure that bridges the viral and cellular membranes into a close proximity for fusion. Different from other classes of anti-HIV drugs such as inhibitors of reverse transcriptase and protease that act after infection occurs, HIV-1 entry inhibitors intercept the virus before it invades the target cells. Currently, there are two HIV-1 entry inhibitors for clinical use: Maraviroc binds to the cell coreceptor CCR5 thus blocking the binding of virus [3,4]; Enfuvirtide (T20), a 36-amino acid peptide derived from the gp41 CHR, binds competitively to the NHR thus preventing the formation of 6-HB core [5-7]. Approved in April 2003, T20 is the first and only HIV-1 fusion inhibitor used in clinic but it requires a high dosage and easily induces drug-resistance [8-11], calling for new strategies and concepts for the development of next-generation drugs targeting HIV fusion.

The crystal structure of 6-HB core reveals a deep hydrophobic pocket on the C-terminal portion of NHR trimer, which is inserted by three hydrophobic residues from the pocket-binding domain (PBD) of CHR [12-14]. It is believed that the pocket critically determines the stability of NHR-CHR interaction and can serve as an ideal target for inhibitors [15,16]. Due to the lack of the pocket-binding sites by T20, the CHR-derived peptide C34 has been widely used as a template for peptide engineering [17-19]. As a key strategy, the salt-bridge structures were introduced into C34 sequence creating the electrostatically constrained peptides such as SC34EK [20], T2635 [21] and Sifuvirtide (SFT) [22], in which the amino acids at the solvent-accessible sites of helical bundle were replaced with glutamate (E) and lysine (K) and those at the NHR-interactive sites were maintained, thus in an α-helical heptad repeat residues separated by three positions (*i* versus *i* + 4) were closely positioned in space on the same site of the helix (Figure 1). As compared to C34, these electrostatically-engineered inhibitors possessed the significantly improved anti-HIV profiles [20-22]. By truncating the C-terminus of SC34EK, the relatively short peptide SC29EK was generated with a comparable anti-HIV activity but its further truncation (SC22EK) could not be tolerated [20]. Recently, we discovered that two residues (Met115 and Thr116) preceding the pocket-binding domain of CHR peptides adopt a unique M-T hook structure that can greatly enhance the pocket-binding [23]. Indeed, the M-T hook structure-modified C34 and SC22EK exhibited the dramatically increased binding affinity and antiviral activity [24,25], suggesting a totally new strategy for designing or optimizing HIV-1 fusion inhibitors. In this study, we applied two hook residues to modify SC29EK and observed a significant optimization. Importantly, the resulting peptide MT-SC29EK showed a highly improved potency to inhibit T20- and SC29EK-resistant HIV-1 variants and a higher genetic barrier to resistance. Our studies have validated a general feature of the M-T hook structure for designing HIV-1 fusion inhibitors and offered a promising candidate for future development.

## Results

### The M-T hook residues dramatically enhance the stability of 6-HB core

**Figure 2:**
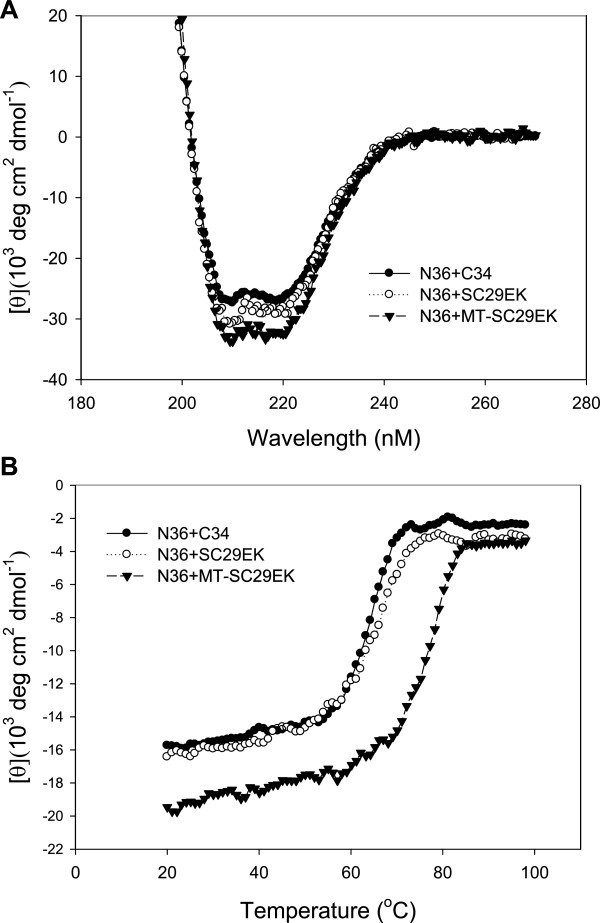
**Helical stability of SC29EK and MT-SC29EK determined by CD spectroscopy.** The α-helicity **(A)** and thermostability **(B)** of 6-HB formed by a CHR peptide (SC29EK or SC29EK) and the NHR peptide N36 are shown. Final concentration of each peptide in PBS is 10 μM.

To validate a general role of the M-T hook structure and develop a more active HIV-1 fusion inhibitor, we generated the peptide MT-SC29EK by adding the residues methionine (Met115) and thronine (Thr116) into the N-terminus of SC29EK. The CD spectroscopy was first applied to determine whether two hook residues can enhance the α-helicity and thermal stability of the 6-HB structure. A CHR peptide (C34, SC29EK or MT-SC29EK) was mixed with the NHR peptide N36 at equal molar concentrations and incubated at 37°C for 30 min. As showed in Figure 2A, the CD spectra of all three peptide pairs displayed typical double minima at 208 and 222 nm, indicating the formation of the α–helical secondary structures. Obviously, the α-helicity of MT-SC29EK/N36 complex slightly increased as compared to that of SC29EK/N36 and C34/N36 complexes. The thermostability of each 6-HBs, defined as the midpoint of the thermal unfolding transition (*T*_*m*_) value, was further measured. As shown in Figure 2B, SC29EK-based 6-HB had a significantly increased *T*_*m*_ value (69.1°C) relative to the C34-based 6-HB (65.0°C), but the *T*_*m*_ value of MT-SC29EK-based 6-HB was dramatically increased to 79.1°C, indicating that addition of two M-T hook residues can dramatically enhance the thermal stability of 6-HB conformation.

### High-affinity interactions of MT-SC29EK with the NHR target

**Figure 3:**
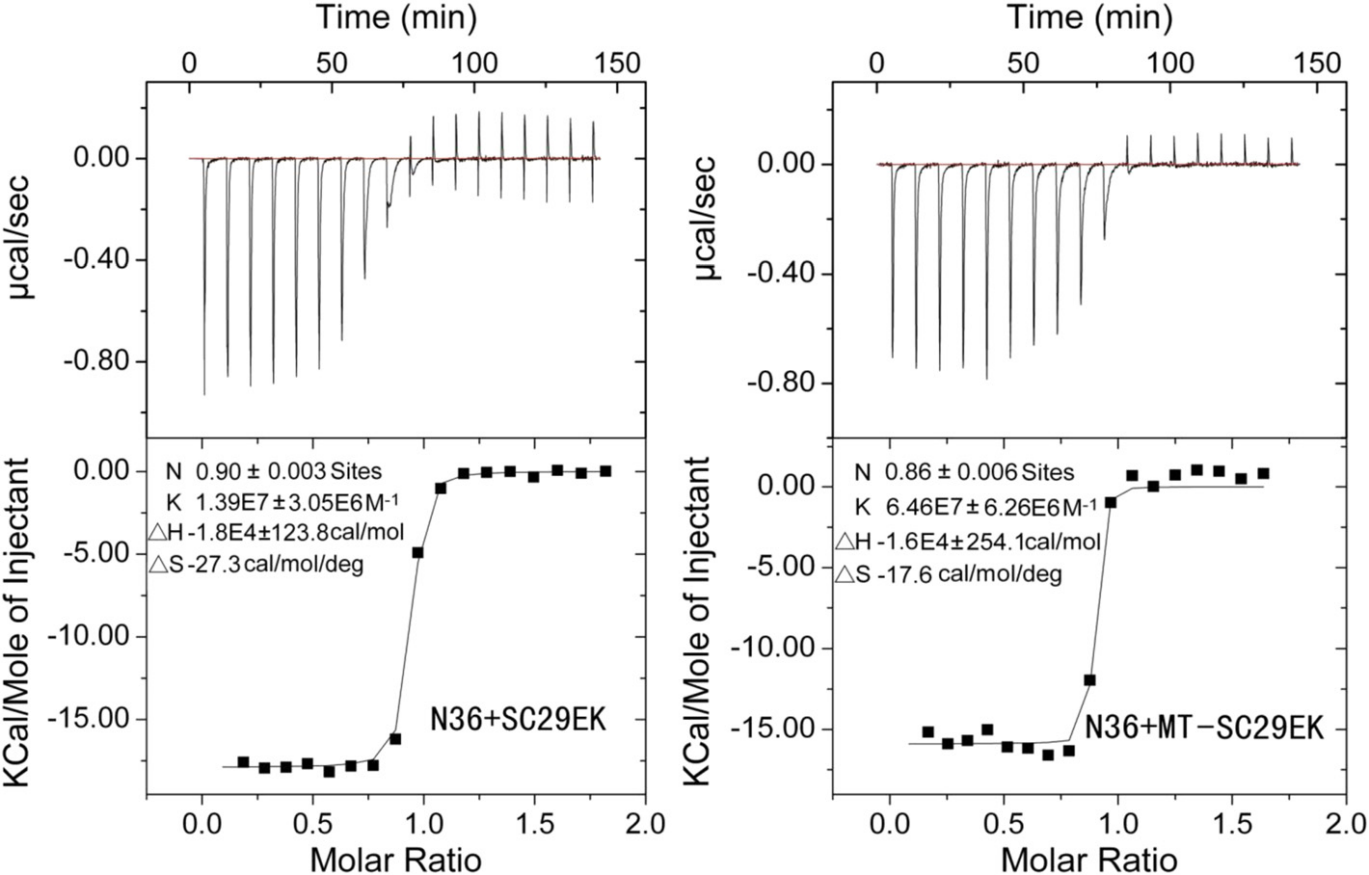
**Interaction affinity of SC29EK and MT-SC29EK with N36 measured by ITC assay.** 1 mM N36 dissolved in double distilled H2O was injected into the chamber containing 100 μM SC29EK (Left) or MT-SC29EK (Right). The experiments were carried out at 25°C. Data acquisition and analysis were performed using MicroCal Origin software (version 7.0). The upper panels show the titration traces and the lower panels show the binding affinity when the N36 solution was injected into the SC29EK or MT-SC29EK solution.

We then used the ITC technology to determine the thermodynamic profiles of the molecular interaction between the inhibitors and N36. The heat released or absorbed during the interaction allowed an accurate determination of the binding constant (K), reaction stoichiometry (N), enthalpy (ΔH) and entropy (ΔS). As shown in Figure 3, the formation of 6-HBs formed by SC29EK and N36 or MT-SC29EK and N36 is a typical enthalpy-driven reaction, in which a large amount of heat is released. Compared to SC29EK, the K value of MT-SC29EK increased 4.7-folds (from 1.39 × 10^7^ to 6.46 × 10^7^ M^-1^), suggesting a dramatically increased interacting-affinity. Together with the CD data, we confirmed that the M-T hook residues can dramatically fortify the binding affinity of peptide inhibitors.

### The M-T hook residues markedly increase the potency of SC29EK to block 6-HB

**Figure 4:**
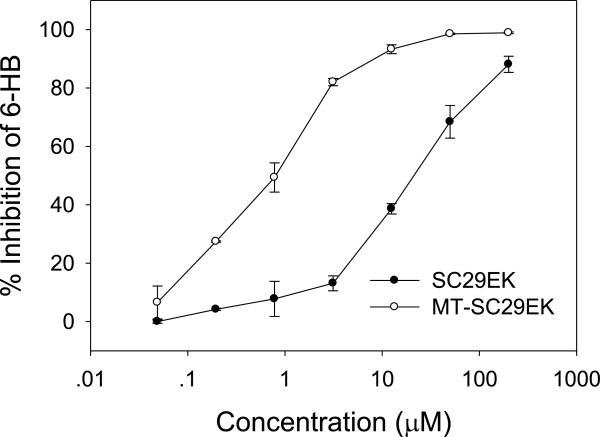
**Inhibition of SC29EK and MT-SC29EK on 6-HB as measured by ELISA.** Data were derived from the results of 3 independent experiments and are expressed as means ± S.D.

Inhibition of CHR peptides on HIV-1 entry is through binding to the exposed NHR thus competing off the viral CHR, namely blocking 6-HB formation during the fusion reaction. We previously developed a capture ELISA-based approach to screen or evaluate the peptide or small molecule-based fusion inhibitors [26], in which the 6-HB-specific antibody NC-1 was coated to the ELISA plate as a capture and the biotinylated-C34 was used for a signal detection. Here, we compared the potency of SC29EK and MT-SC29EK to physically block the 6-HB formation. As shown in Figure 4, both peptides were able to block the 6-HB at a dose dependant manner, however, SC29EK had an IC_50_ of 14 μM whereas MT-SC29EK had an IC_50_ value of 0.6 μM, indicating a 23-fold increase. Therefore, the simple addition of two M-T hook residues did confer the inhibitors with a high strength to target the NHR, in line with the biophysical data described above.

### The M-T hook residues significantly improve the antiviral activity of SC29EK

**Figure 5:**
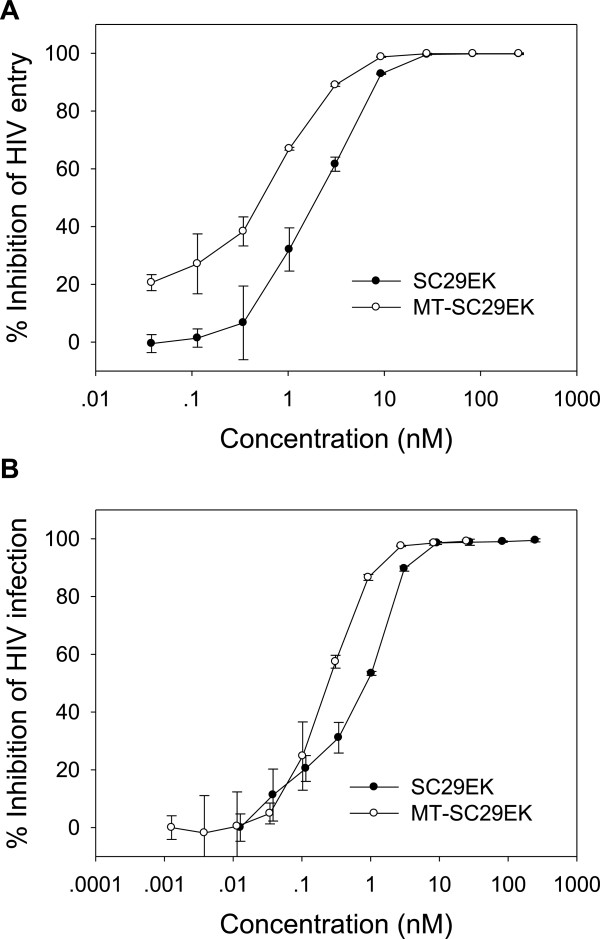
**Anti-HIV activity of SC29EK and MT-SC29EK. (A)** Inhibition of HIV-1_NL4–3_ pseudovirus entry. **(B)** Inhibition of the wild-type HIV-1_NL4–3_ replication. Data were derived from the results of 3 independent experiments and are expressed as means ± S.D.

Based on its high binding to the NHR target and potent inhibition on the 6-HB formation, we expected that MT-SC29EK has an increased antiviral activity as compared to SC29EK. In the single-cycle entry assay (Figure 5A), SC29EK and MT-SC29EK inhibited HIV-1_NL4–3_ pseudoviruses with IC_50_ values of 2.2 ± 0.3 nM and 0.7 ± 0.0 nM respectively, indicating a 3.1-fold increased potency for MT-SC29EK. For the wild-type HIV-1_NL4–3_ replication (Figure 5B), SC29EK had an IC_50_ of 0.9 ± 0.1 nM, whereas MT-SC29EK had an IC_50_ of 0.2 ± 0.0 nM, indicating a 4.5-fold increase. These results indicated that two M-T hook residues can significantly enhance the anti-HIV activity of SC29EK. In parallel, we also tested a panel of well-known control peptides, including T20, C34, SFT, CP32M, T1249 and T2635 (Table 1). Promisingly, MT-SC29EK showed the most potent inhibitory activity against HIV-1 fusion and infection, highlighting its potential for clinical development.

**Table 1:** **Anti-HIV activity of MT-SC29EK and control peptides**^**a**^

		**IC50 (nM)**	
**Inhibitor**	**Length (a.a.)**	**Entry**	**Replication**
T20	36	73.2 ± 9.1	48.6 ± 1.2
C34	34	2.7 ± 0.1	1.3 ± 0.2
SFT	36	2.6 ± 0.1	1.5 ± 0.1
CP32M	32	1.8 ± 0.2	1.0 ± 0.2
T1249	39	1.4 ± 0.2	1.2 ± 0.2
T2635	38	1.1 ± 0.1	0.4 + 0.2
SC22EK	22	51.3 + 5.4	58.2 + 15.2
MT-SC22EK	24	2.1 ± 0.2	1.1 ± 0.1
SC29EK	29	2.2 ± 0.3	0.9 ± 0.2
MT-SC29EK	31	0.7 ± 0.0	0.2 ± 0.0

^a^The data were derived from the results of three independent experiments and expressed as means ± SD; a.a. means the number of amino acids.

### Potent activity of MT-SC29EK against T20- and SC29EK-resistant HIV-1 variants

The mutations of T20-resistance have been largely mapped to the peptide binding region in the NHR of gp41 [8,10,27]. We have recently showed that the M-T hook residues can dramatically improve the potency of CHR peptides to inhibit T20-resistant HIV-1 variants, which carry single or double T20-resistant mutations [24,25]. Here, we compared the inhibitory activity of SC29EK and MT-SC29EK for those HIV-1 mutants (Table 2). As shown in Table 2, all the tested T20-resistant HIV-1 variants conferred high cross-resistance to SC29EK; however, MT-SC29EK retained high potency against these T20- and SC29EK- resistant viruses. Notably, the single Q40H mutation resulted in >25.4-fold resistance for T20 and 46.6-fold resistance for SC29EK, but it did not affect the inhibitory activity of MT-SC29EK. The similar phenotypes were also observed for the I37T and V38A single mutations and the V38A/Q42T double mutation, which rendered high resistance to T20 and SC29EK but were highly sensitive to MT-SC29EK. As compared to SC29EK, the inhibitory activity of MT-SC29EK on various HIV-1 variants dramatically increased.

**Table 2:** **Inhibitory activity of SC29EK and MT-SC29EK against T20-resistant HIV-1 variants**^**a**^

	**T20**		**SC29EK**		**MT-SC29EK**	
**HIV-1**_**NL4–3**_	**IC**_**50**_	**n-fold**^**b**^	**IC**_**50**_	**n-fold**	**IC50**	**n-fold**
WT	52.1 ± 8.5	1	1.8 ± 0.2	1	0.6 ± 0.1	1
I37T	552.2 ± 123.4	10.6	15.0 ± 0.3	8.3	0.8 ± 0.2	1.3
V38A	1842.6 ± 56.1	35.4	16.2 ± 1.9	9	0.6 ± 0.1	1
V38M	378.3 ± 41.7	7.3	15.1 ± 1.8	8.4	1.7 ± 0.0	2.8
Q40H	1321.5 ± 92.3	25.4	83.8 ± 10.8	46.6	0.8 ± 0.1	1.3
N43K	366.8 ± 47.4	7	>250	>138.9	7.2 ± 0.2	12
D36S/V38M	202.6 ± 12.6	3.9	11.7 ± 0.7	6.5	1.2 ± 0.0	2
I37T/N43K	>2250	>43	>250	>138.9	21.2 ± 4.4	35.3
V38A/N42T	>2250	>43	37.7 ± 6.4	20.9	0.6 ± 0.0	1

^a^The data were derived from the results of three independent experiments and expressed as means ± SD. ^b^*n*-fold values are relative to the IC50 of the wild-type (WT) virus.

### High-affinity binding of MT-SC29EK to the NHR mutants

**Figure 6:**
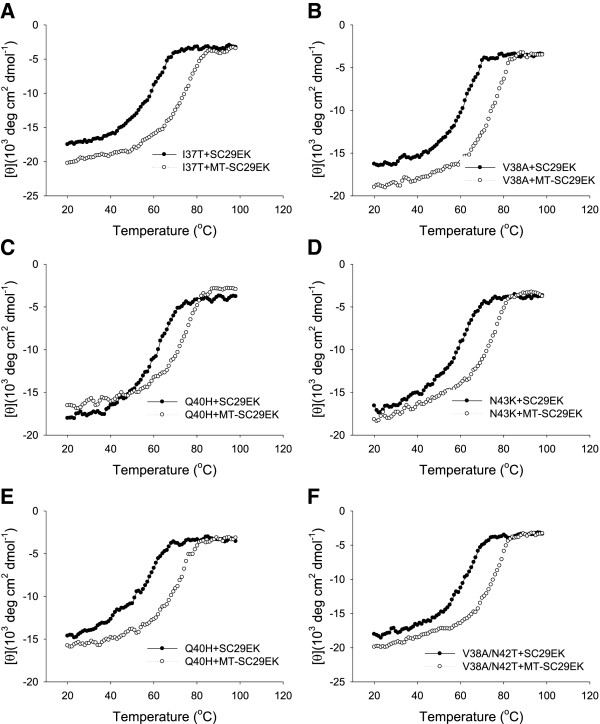
**Binding stability of SC29EK and MT-SC29EK with N36 mutants determined by CD spectroscopy. (A)** I37T; **(B)** V38A; **(C)** Q40H; **(D)** N43K; **(E)** I37T/N43K; **(F)** V38A/N42T. Final concentration of each peptide in PBS is 10 μM.

To get insights how the M-T hook-modified inhibitors possess high potency against drug-resistant HIV-1 variants, we continued to compare the binding affinity of SC29EK and MT-SC29EK to the NHR mutants. A panel of N36-based peptides carrying a single (I37T, V38A, Q40H, N43K) or double (I37T/N43K and V38A/N42T) NHR mutations were applied in the CD analysis. In most cases, the NHR mutations resulted in an obvious decrease in the thermal stability of SC29EK-based 6-HBs, such as V38A (*T*_*m*_ = 62.1°C) and I37T/N43K (*T*_*m*_ = 61.1°C) mutants, but addition of two hook residues could largely recover the loss (Figure 6). In other words, the *T*_*m*_ values of MT-SC29EK-based 6-HBs dramatically increased. Therefore, the M-T hook residues could compensate the binding affinity in the NHR mutants thus preserving the high inhibitory activity against SC29EK- and T20-resistant HIV-1 variants.

### MT-SC29EK displays a high genetic barrier to the development of resistance

**Figure 7:**
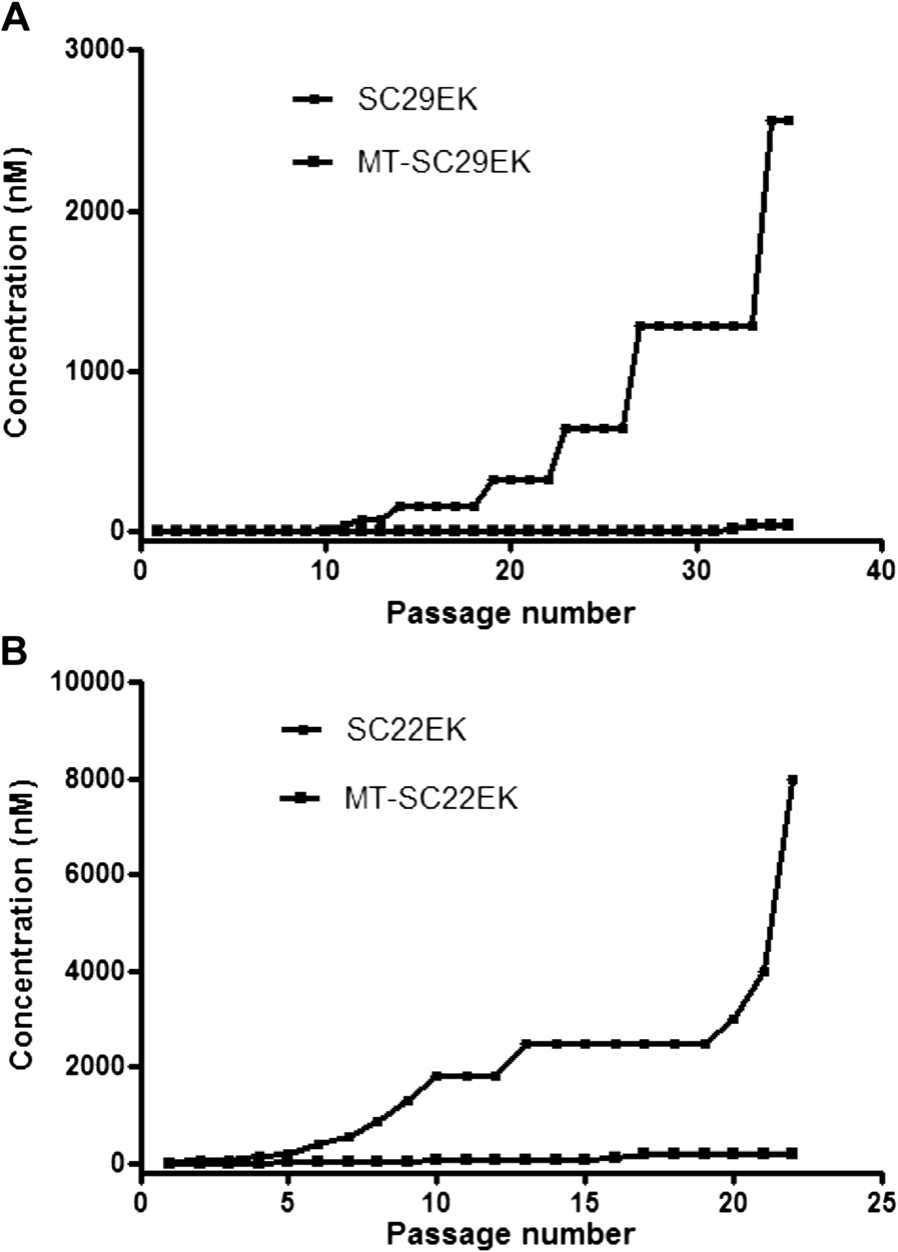
***In vitro *selection of HIV-1 resistant to fusion inhibitors. (A)** Induction of resistant HIV-1 with SC29EK and MT-SC29EK; **(B)** Induction of resistant HIV-1 with SC22EK and MT-SC22EK. The molecular clone of HIV-1_NL4–3_ was passaged in the presence of 1.5-2 fold escalating concentrations of peptides. One culture is shown as a representative.

We asked whether the M-T hook residues can confer the inhibitors with a high genetic barrier to the development of resistance. To address this, we performed the *in vitro* selection of resistant HIV-1_NL4–3_ for SC29EK and MT-SC29EK in parallel. As shown in Figure 7A, the virus could be gradually passaged in the presence of dose-escalating SC29EK. After 35 generations of passage over 7 months, the concentration of SC29EK was raised to 2,650 nM; in a sharp contrast, virus escape from inhibition by the MT-SC29EK peptide was much more difficult than escape from the SC29EK peptide.We were surprised that it was much more difficult to select viruses resistant to the M-T hook-modified SC29EK inhibitor compared to unmodified inhibitor. To prove this finding, we compared the short-peptide pair SC22EK and MT-SC22EK for inducing the resistance, as our previous studies demonstrated that the MT-SC22EK possessed a significantly improved activity than SC22EK to inhibit both wild-type and T20-resistant HIV-1 strains. Consistently, the virus culture could be reasonably recovered after 22 passages in 4 months, wherein the concentration of SC22EK was escalated up to as high as 8,000 nM (Figure 7B). However, MT-SC22EK only approached to 100 nM due to the difficulty of the virus passage. These results implied that the M-T hook-modified peptide inhibitors might possess a dramatically increased genetic barrier to the development of resistance.

## Discussion

Previously, we identified that the heptad amino acid motif (^110^QIWNNMT^116^) preceding the pocket-binding domain of CHR-derived peptides could dramatically enhance the binding affinity and antiviral activity of inhibitors [28]. Based on the QIWNNMT motif-containing peptide CP621-652, we developed a potent HIV-1 fusion inhibitor named CP32M [29]. However, we did not know the molecular determinants underlying the stability and anti-HIV activity of inhibitors in detail. Recently, we solved the high-resolution crystal structure of CP621-652 complexed by a NHR-derived peptide (T21) [23]. Surprisingly, we found the M-T hook structure, in which the residue Thr116 redirects the peptide chain to position Met115 above the left side of the hydrophobic pocket on the NHR trimer and the side chain of Met115 caps the hydrophobic pocket to stabilize the interaction between the pocket and the pocket-binding domain [23]. To directly define the structure and function of the M-T hook, we generated the peptide MT-C34 by incorporating Met115 and Thr116 into the N-terminus of C34 [24]. The high resolution crystal structure of MT-C34 verified a universal structural feature for CHR-based inhibitors. We also demonstrated that addition of two hook residues could dramatically enhance the binding affinity and thermal stability of 6-HB core. Compared with C34, MT-C34 exhibited the significantly increased activity to inhibit HIV-1 fusion and replication [24]. These findings prompted us to propose a new strategy for designing HIV-1 fusion inhibitors. Very recently, we verified this concept by introducing the M-T hook structure into several CHR-derived short peptides and observed a significant optimization [25]. In the present study, we selected SC29EK as a template to validate the general role of the M-T hook structure, as this electrostatically constrained helical peptide has a relatively short sequence but possesses a potent antiviral activity thus having potential to be further developed for clinical use [20].

The initial structural data of gp41 6-HBs informed the mechanisms of HIV-1 fusion and its inhibition [12-14]. The CHR-derived peptides act by competitive binding to the exposed NHR during its conformational change to the fusogenic state (*i.e.* pre-hairpin conformation) and thus block the 6-HB formation in a dominant-negative fashion [16,17]. It is generally thought that a high-affinity binding is required for an exogenous peptide to compete off the viral CHR thus critically determining the antiviral activity, thereby a number of strategies have been explored to improve the binding affinity of inhibitors [11,17-19,30]. Our series of studies have provided a new approach that can dramatically enhance the binding of inhibitors to the NHR target [23-25,28,29]. The biophysical data presented here indicated that the binding stability of MT-SC29EK could be greatly increased as compared to that of SC29EK. It is conceivable that two M-T hook residues may integrate the pocket-binding domain thus synergistically enhancing the interactions of MT-SC29EK with the targeting NHR region. Consistent to this hypothesis, MT-SC29EK could physically block the 6-HB formation more efficiently and possessed higher anti-HIV activity. Promisingly, MT-SC29EK showed the most potent inhibition against HIV-1 entry and replication as compared to the well-characterized first- (T20, C34) and next- (T1249, SFT, T2635) generations of HIV-1 inhibitors.

Like other classes of anti-HIV drugs, HIV-1 fusion inhibitors are also facing the problem of drug-resistance. The emergence and spread of T20-resistant HIV-1 strains have already resulted in increased number of patients failing to treatment. T20-resistance has been predominantly mapped to the substitutions in the amino acid 36–45 of the NHR, with a contiguous three residues (G36-I37-V38) being a hotspot [8-10,31,32]. Similar to SC34EK and SC29EK, Sifuvirtide (SFT) was also designed by introducing multiple salt-bridges into C34 [22]. Due to its potent anti-HIV activity, SFT has already been advanced into Phase III clinical trials in China and may become the second HIV-1 fusion inhibitor for clinical use. However, SFT could easily induce drug-resistance in the *in vitro* selection and HIV-1 variants displayed high cross-resistance to T20 [33]. The *in vitro* selection and resistant profiles of SC34EK and T2635 were also reported [34,35]. Eggink *et al.*[36] described four mechanisms of drug resistance: reduced contact, steric obstruction, electrostatic repulsion, and electrostatic attraction. From SFT-resistance, we deduced several additional mechanisms, such as hydrogen bond disruption and hydrophobic contact disruption [33,37]. Therefore, an ideal next-generation HIV-1 fusion inhibitor should efficiently inhibit the existing inhibitor-resistant HIV-1 variants but itself possessing a high genetic barrier to overcome drug-resistance. Our studies suggested that the M-T hook structure might confer the inhibitors these two features. First, MT-SC29EK was able to potently inhibit a panel of major T20-resistant HIV-1 variants, which also displayed high-level cross-resistance to SC29EK. More impressively, MT-SC29EK and its short version MT-SC22EK suffered from the difficulty of inducing drug-resistance during the *in vitro* selection, implying their considerable higher genetic barriers than SC29EK or SC22EK to the development of resistance. Very recently, we have found that the M-T hook-modified C34 (MT-C34) and Sifuvirtide (MT-SFT) behaved with a similar phenotype (unpublished data). Taken together, we consider that the M-T hook structure can render the peptide fusion inhibitors with a high genetic barrier to select the resistant HIV-1 variants, which provides an important feature for drug development. It is well established that the residues involving interactions in the NHR pocket are highly conserved during HIV-1 evolution; the mutations of these residues are often lethal to the virus. Thus, the balance between virus survival and drug resistance becomes hard to maintain, and the genetic barrier for drug resistance against the M-T hook should increase [2,15]. Furthermore, it has been shown that if the drug-target affinity is too high, the virus does not escape by mutating the binding site, but by reducing the window of opportunity for the drug to act [19,21,35]. Definitely, more follow-up studies are required to explore how the M-T hook structure can dramatically increase the potency of inhibitors against the known resistant HIV-1 variants and simultaneously confer a high genetic barrier to resistance. Besides selecting the escape viruses and mapping the responsible mutations, the mechanism of action by the M-T hook structure-modified inhibitors should be pursued in the context of the recently solved Env trimer structure [38-40].

## Conclusions

We demonstrated that two M-T hook residues have greatly improved the antiviral profiles of SC29EK, verifying the M-T hook structure as a general strategy for designing or optimizing HIV-1 fusion inhibitor. The resulting inhibitor MT-SC29EK has potent inhibitory activity against diverse HIV-1 strains and possesses a high genetic barrier to resistance, thus offering a promising candidate for drug development.

## Methods

### Peptide synthesis

A panel of CHR peptides including T20, C34, SFT, CP32M, T1249, T2635, SC29EK, MT-SC29EK, SC22EK, MT-SC22EK and the NHR peptide N36 and its mutants (I37T, V38A, Q40H, N43K, I37T/N43K, and V38A/N42T) were synthesized by a standard solid-phase FMOC method as described previously [24]. All peptides were acetylated at the N-terminus and amidated at the C-terminus and purified by reversed-phase high-performance liquid chromatography (HPLC). They were verified for purity >95% and correct amino acid composition by mass spectrometry. Concentrations of the peptides were determined by UV absorbance and a theoretically calculated molar-extinction coefficient ϵ (280 nm) of 5500 M^-1^ · cm^-1^ and 1490 M^-1^ · cm^-1^ based on the number of tryptophan and tyrosine residues, respectively [41].

### Circular dichroism (CD) spectroscopy

CD spectroscopy was performed according to our protocols described previously [26]. Briefly, a CHR peptide (C34, SC29EK or MT-SC29EK) was incubated with an equal molar concentration of N36 or its mutant (I37T, V38A, Q40H, N43K, I37T/N43K, and V38A/N42T) at 37°C for 30 min. The final concentration of each peptide was 10 μM in PBS buffer (pH 7.2). The CD spectra were acquired on a Jasco spectropolarimeter (model J-815) using a 1 nm bandwidth with a 1 nm step resolution from 195 to 260 nm at room temperature. The spectra were corrected by subtraction of a blank corresponding to the solvent. Data were averaged over three accumulations. The α-helical content was calculated from the CD signal by dividing the mean residue ellipticity [θ] at 222 nm by the value expected for 100% helix formation (-33,000 degrees.cm^2^.dmol^-1^). The thermal denaturation experiment was performed by monitoring the change in ellipticity [θ] at 222 nm at the increasing temperature (20–98°C) using temperature controller. The temperature was increased at a rate of 1.2°C per min; data were acquired at a 1 nm bandwidth at 222 nm at a frequency of 0.25 Hz. The melting curve was smoothened, and the midpoint of the thermal unfolding transition (*T*m) values were taken as the maximum of the derivative d [θ]_222_/dT. The *T*m value was detected at a peptide concentration of 10 μM in PBS buffer.

### Isothermal Titration Calorimetry (ITC)

ITC assay was performed using an ITC_200_ Microcalorimeter instrument (MicroCal, USA) as described previously [24]. In brief, 1 mM N36 dissolved in ddH_2_0 was injected into the chamber containing 100 μM SC29EK or MT-SC29EK. The experiments were carried out at 25°C. The time between injections was 240 s and the stirring speed was 500 rpm. The heats of dilution were determined in control experiments by injecting N36 into ddH_2_0 and subtracted from the heats produced in the corresponding peptide-peptide binding experiments. Data acquisition and analysis were performed using MicroCal Origin software (version 7.0).

### Inhibition of 6-HB formation by peptides

The 6-HB core-specific monoclonal antibody NC-1 was obtained from Dr. Shibo Jiang in the New York Blood Center (New York, NY) through the ARRRP, Division of AIDS, NIAID, National Institute of Health. The inhibitory activity of SC29EK or MT-SC29EK on the 6-HB formation was measured by a modified ELISA as previously described [26]. Briefly, a 96-well polystyrene plate was coated with 2 μg/ml NC-1 in 0.1 M Tris buffer (pH 8.8). A tested peptide at graded concentrations was mixed with the biotinylated-C34 (0.1 μM) and incubated with N36 (0.1 μM) at room temperature for 30 min. The mixture was then added to the NC-1-coated plate, followed by incubation for 30 min and washing with a washing buffer (PBS containing 0.1% Tween 20) three times. Then horseradish peroxidase (HRP)-labeled streptavidin (Invitrogen) and the substrate 3,3,5,5- tetramethylbenzidine (Sigma) were sequentially added. Absorbance at 450 nm (*A*450) was measured using an ELISA reader.

### Measurement of anti-HIV activity of peptides

The inhibition of peptides on the HIV-1 entry was determined by single-cycle infection assay as described previously [42]. Briefly, HIV-1 pseudovirus was generated by cotransfecting 293 T cells with an Env-expressing plasmid and a backbone plasmid pSG3^Δenv^ that encodes Env-defective, luciferase-expressing HIV-1 genome. The supernatants were harvested and filtered 48 h after transfection and 50% tissue culture infectious dose (TCID_50_) was determined in TZM-bl cells. The peptides were prepared with 3-fold dilutions and mixed with 100 TCID_50_ viruses and then incubated 1 h at room temperature. The mixture was added to TZM-bl cells (10^4^/well) and incubated 48 h at 37°C. The luciferase activity was measured using luciferase assay reagents and a Luminescence Counter (Promega).

The inhibition of peptides on HIV-1 replication was determined by a molecular cloned wild-type HIV-1_NL4–3_. In brief, the virus stock was harvested and quantified 48 h post-transfection. 100 TCID_50_ viruses were used to infect TZM-bl cells in the presence or absence of serially diluted peptides. Two days post-infection, the cells were harvested and lysed in reporter lysis buffer and the luciferase activity was measured as described above.

### Induction of HIV-1 resistance to inhibitors

The *in vitro* selection of HIV-1 resistance to peptide inhibitors was performed as described previously [33]. Briefly, MT-4 cells were seeded at 1 × 10^4^ in RPMI 1640 medium containing 10% fetal bovine serum on 12-well plates. The molecular clone of HIV-1_NL4–3_ was used to infect the cells in the presence or absence of diluted peptide inhibitors (SC29EK, MT-SC29EK, SC22EK and MT-SC22EK). The cells were incubated at 37°C with 5% CO_2_ until an extensive cytopathic effect was observed. The culture supernatants were harvested and used for next passage on fresh MT-4 cells with 1.5-2 fold increasing concentrations of peptide. Cells and supernatant were harvested at regular time points and stored at -80°C.

## Competing interests

The authors declare that they have no competing interests.

## Authors’ contributions

HC, ZQ, JS, YQ, XL carried out the experiments. HC, ZQ analyzed the data. YH conceived and designed the study and drafted the manuscript. All authors read and approved the final manuscript.
